# A Multicenter Observational Cohort Study to Evaluate the Effects of Bisphosphonate Exposure on Bone Mineral Density and Other Health Outcomes in Osteogenesis Imperfecta

**DOI:** 10.1002/jbm4.10118

**Published:** 2019-01-07

**Authors:** Jaskaran S Bains, Erin M Carter, Kate P Citron, Adele L Boskey, Jay R Shapiro, Robert D Steiner, Peter A Smith, Michael B Bober, Tracy Hart, David Cuthbertson, Jeff Krischer, Peter H Byers, Melanie Pepin, Michaela Durigova, Francis H Glorieux, Frank Rauch, Joseph M Sliepka, V Reid Sutton, Brendan Lee, Members of the BBD Consortium, Sandesh CS Nagamani, Cathleen L Raggio

**Affiliations:** ^1^ Hospital for Special Surgery Dept of Orthopedic Surgery New York NY USA; ^2^ Department of Bone and Osteogenesis Imperfecta Kennedy Krieger Institute Baltimore MD USA; ^3^ Departments of Pediatrics and Molecular and Medical Genetics Oregon Health & Science University Portland OR USA; ^4^ University of Wisconsin School of Medicine and Public Health Madison WI USA; ^5^ Shriners Hospitals for Children Chicago IL USA; ^6^ Division of Medical Genetics Alfred I. DuPont Hospital for Children Wilmington DE USA; ^7^ Osteogenesis Imperfecta Foundation Gaithersburg MD USA; ^8^ College of Medicine University of South Florida, Biostatistics Tampa FL USA; ^9^ Departments of Medicine and Pathology Division of Medical Genetics University of Washington Seattle WA USA; ^10^ Shriners Hospital for Children‐Canada and McGill University, Division of Endocrinology Montreal QC Canada; ^11^ Department of Molecular and Human Genetics Baylor College of Medicine Houston TX USA; ^12^ Texas Children's Hospital, Human Genetics Houston TX USA

**Keywords:** OSTEOGENESIS IMPERFECTA, BISPHOSPHONATE, ANTIRESORPTIVE, FRACTURE RISK, RARE BONE DISEASE

## Abstract

Osteogenesis imperfecta (OI) is characterized by low bone mass and bone fragility. Using data from a large cohort of individuals with OI from the Osteogenesis Imperfecta Foundation's linked clinical research centers, we examined the association between exposure to bisphosphonate (BPN) treatment (past or present) and lumbar spine (LS) areal bone mineral density (aBMD), fractures, scoliosis, and mobility. From 466 individuals, we obtained 1394 participant‐age LS aBMD data points. Though all OI subtypes were examined, primary analyses were restricted to type I OI (OI‐1). Using linear regression, we constructed expected OI‐1 LS aBMD‐for‐age curves from the data from individuals who had never received BPN. LS aBMD in those who had been exposed to BPN was then compared with the computed expected aBMD. BPN exposure in preadolescent years (age <14 years) was associated with a LS aBMD that was 9% more than the expected computed values in BPN‐naïve individuals (*p* < 0.01); however, such association was not observed across all ages. Exposure to i.v. BPN and treatment duration >2 years correlated with LS aBMD in preadolescent individuals. BPN exposure also had a significant association with non‐aBMD clinical outcome variables. Logistic regression modeling predicted that with BPN exposure, a 1‐year increase in age would be associated with an 8.2% decrease in fracture probability for preadolescent individuals with OI‐1, compared with no decrease in individuals who had never received any BPN (*p* < 0.05). In preadolescent individuals with OI‐1, a 0.1 g/cm^2^ increase in LS aBMD was associated with a 10.6% decrease in scoliosis probability, compared with a 46.8% increase in the BPN‐naïve group (*p* < 0.01). For the same changes in age and LS aBMD in preadolescent individuals, BPN exposure was also associated with higher mobility scores (*p* < 0.01), demonstrating that BPN treatment may be associated with daily function. © 2018 The Authors. *JBMR Plus* Published by Wiley Periodicals, Inc. on behalf of American Society for Bone and Mineral Research.

## Introduction

Osteogenesis imperfecta (OI), otherwise known as brittle bone disease, is a Mendelian disorder characterized by low bone mass and bone fragility.^1^ The majority of individuals with OI have pathogenic variants in *COL1A1* or *COL1A2*, which encode for α1 and α2 chains of type I collagen, respectively.^2^ Although advances in identifying the genetic bases of OI over the past 15 years have greatly expanded the genetic heterogeneity of OI, most individuals can still be classified into the 1979 Sillence clinical classification of mild (type I), perinatally lethal (type II), severe (type III), and moderate (type IV) forms.^2^, ^3^, ^4^ Most individuals with type I OI (OI‐1) have haploinsufficiency of type I collagen, whereas the majority with types II, III, and IV OI have a qualitative abnormality of type I collagen.^5^

Bisphosphonates (BPNs), a class of drugs that inhibit osteoclast function and decrease bone resorption, are commonly used to treat OI.^6^, ^7^, ^8^, ^9^, ^10^, ^11^ Independent clinical studies, most of which were conducted in relatively small populations, have shown an association between BPN treatment and improved clinical outcomes, including decreased fracture rates^12^, ^14^, ^15^ and improved mobility.^12^, ^13^, ^14^ However, recent meta‐analyses have not demonstrated conclusive evidence that BPNs decrease fracture risk or lead to improvement in clinical outcomes in OI.^16^, ^17^, ^18^

In this study, we used data from the Osteogenesis Imperfecta Foundation's linked clinical research centers (LCRCs) to assess the effect of ever‐exposure to BPN (past or present) on lumbar spine (LS), areal bone mineral density (aBMD), fractures, scoliosis, and mobility in individuals with OI. The LCRCs were composed of clinical centers in the United States and Canada, a data management coordinating center, and a center for molecular and biochemical analysis.^5^, ^19^ This group collaborated to conduct a longitudinal study of osteogenesis imperfecta, a large observational study that enrolled 551 individuals with various types of OI. We analyzed these data to investigate whether BPN exposure was associated with: (1) an increase in LS aBMD; (2) a decrease in fracture probability and number of fractures; (3) a decrease in occurrence and severity of scoliosis; and (4) an increase in mobility as compared with individuals not treated with BPNs within the same OI subtype.

## Subjects and Methods

### Subjects

Informed consent and assent (as appropriate) were obtained from each participant. The research protocol was approved by the institutional review board at each site. Data were collected at the following sites of the LCRCs: Oregon Health & Science University (Portland, OR, USA), Kennedy Krieger Institute (Baltimore, MD, USA), Baylor College of Medicine (Houston, TX, USA), Shriners Hospital for Children (Chicago, IL, USA), Nemours/Alfred I. duPont Hospital for Children (Wilmington, DE, USA), and the Shriners Hospital for Children (Montreal, QC, Canada). The study data were collected between 2009 and 2014. Individuals with a clinical, molecular, or biochemical diagnosis of OI were enrolled in the study. For those without a molecular or biochemical diagnosis, the site's principal investigator and one of the two project principal investigators were required to agree about the clinical diagnosis and subtype of OI based upon specific criteria outlined in the Manual of Operations for the LCRCs (Supplemental Table 1). The exclusion criteria were: (1) individuals without a confirmed diagnosis of OI; (2) individuals with OI and second genetic or syndromic condition; (3) inability to return for annual visits; and (4) individuals with a diagnosis of other skeletal dysplasias. Whereas the primary classification into OI types was based on clinical features, genotypic information, when available, was used to appropriately reclassify patients. Over 90% of individuals enrolled in the study had type I collagen‐related OI, which is generally representative of the proportion in the overall population of individuals with OI.

Data were collected on 551 individuals by visits conducted on an annual basis as previously described.^19^ Data on age, BPN exposure, and OI subtype were available on 478 individuals who were included in the analyses. Participant‐level data on BPN exposure included: (1) a binary variable for whether an individual was ever treated with BPN; (2) type of BPN (oral versus i.v.); (3) one or more LS aBMD measurements; and (4) duration of BPN exposure. The information regarding start and end dates for BPN exposure was not available. From these 478 individuals, we obtained 1479 participant‐age data points. LS aBMD measurements were available for 1394 of these data points from 466 subjects. Individuals with OI‐1 (219 patients) comprised 670 of these data points. Patients of all OI types from ages 0 to 14 years (preadolescent subjects) made up 762 data points.

Analyses were separated by OI type, as different types present varying progressions of LS aBMD with age, as well as distinct fracture probabilities and scoliosis patterns. Ninety‐four percent of patient‐age data points were from participants with type I collagen‐related OI: OI‐1, OI‐3, or OI‐4. OI‐1 comprised 48% of the cohort. Whereas 86% of participants with OI‐3 and 92% of participants with OI‐4 had been treated with BPNs, only 50% of OI‐1 participants had a history of exposure to BPNs (Table 1). The rationale for initiating BPN therapy was also not available. Because of the number of individuals with OI‐1 in the cohort and the even proportion of treated and untreated OI‐1 participants, the principal analyses detailed below were restricted to OI‐1. Whereas the exact start and stop dates of BPN treatment were not available, information on the duration of exposure was available for both oral and i.v. BPN was recorded. Thirty‐seven of the 466 patients (comprising 110 of 1394 data points) received both oral and i.v. BPN treatment and were counted in both the oral BPN‐ and i.v. BPN‐exposure groups. There was no information on whether oral and i.v. BPN treatment overlapped; thus, a cumulative treatment duration could not be computed.

**Table 1:** Individuals with OI Enrolled in the LCRC and Status of BPN Treatment

OI subtype	Number of patients	Treated %	Number of treated data points
I	219	50	337
II	3	100	8
III	79	92	206
IV	139	86	380
V	13	69	30
VI	8	100	29
VII	5	100	13
Total	466	70	1003

OI‐1 = osteogenesis imperfecta; LCRC = Osteogenesis Imperfecta Foundation's linked clinical research centers; BPN = bisphosphonate.

Each site provided LS aBMD measurements as assessed by DXA. All participants had a DXA scan performed on an annual basis. Longitudinal LS aBMD measurements for individual participants were collected using DXA machines at the local sites. The *Z*‐scores were generated by the manufacturer‐provided protocols; there was no centralized reading of the data.

Data were also collected on a variety of clinical outcomes. Self‐reported fractures were recorded during the research visits, as were the type and number of fractures. Information about presence or absence of scoliosis and degree of scoliosis was recorded. The diagnosis of scoliosis was not standardized. Some participants were diagnosed with radiographs ordered as part of their normal clinical care. However, radiographs were not obtained as a part of this study, so some patients with scoliosis were identified via medical records or self‐reporting. Information on mobility was collected using a functional mobility scale. An individual's ability to walk 5 meters, 50 meters, and 500 meters was estimated on a scale of 1 to 6 as follows: 1 = uses wheelchair, 2 = uses walker/frame, 3 = uses crutches, 4 = uses sticks, 5 = independent on level surfaces, and 6 = independent on all surfaces. The sum of these three scores comprised the functional mobility score (0 to 18).^20^

All data were collected in a standardized form across all sites according to the Manual of Operations for the LCRCs. Data quality was assured across sites by the University of South Florida, the Data Management and Coordinating Center of the Rare Diseases Clinical Research Network.

### Statistical analyses

LS aBMD analyses rested on the assumptions that: (1) LS aBMD increases at a diminishing rate over time as a function of age until adulthood; (2) LS aBMD in OI is lower than in the general population; and (3) LS aBMD progression with age would differ based on OI type.

We generated expected LS aBMD versus age curves in participants with OI‐1 who had never been exposed to BPNs using univariate linear regression models. Whereas there was a strong linear relationship between LS aBMD and age in preadolescent years (ages 0 to 14 years), as expected, this effect was not seen over the whole age range of the cohort as peak bone mass acquisition occurs by the third decade of life. Hence, age was log‐transformed before use in regression models using the entire cohort.

The percentage over the expected LS aBMD was then calculated for each OI‐1 participant‐age data point in individuals who had been exposed to BPNs by computing the LS aBMD as a percentage of the observed LS aBMD by the expected LS aBMD curve. A one‐sample, two‐tailed *t* test was performed to compare the distribution of percentages to 100% of the expected mean percentage for the untreated group. This analysis was also conducted separately to determine the differential effects of: (1) i.v. versus oral BPN; (2) exposure duration of less than 24 months versus more than 24 months; and (3) exposure in males versus females. The robustness of these analyses was further verified by using two alternative methods. First, quantile–quantile plots of observed versus expected LS aBMD values for all participants who had been treated with BPNs were created to test for skewness. Second, linear regression analyses of LS aBMD on age, BPN exposure, and the interaction between age and BPN exposure were performed to test the hypothesis that BPN exposure was associated with increased LS aBMD over time as compared with participants who had never received BPNs. Whereas the principal analyses were conducted in OI‐1, the effects of BPN exposure on LS aBMD were also examined in other OI subtypes. LS aBMD *Z*‐scores were separated by subtype and used in a two‐sample *t* test to determine whether the means in the untreated and treated group differed significantly.

Outcome variables other than LS aBMD—fracture probability, fracture number, scoliosis occurrence, scoliosis degree, and mobility score—were analyzed with regression analysis. Fracture probability was not a lifetime measure. Instead, the presence of fractures was assessed for each patient‐age data point and represented with a binary variable. Thus, fracture probability could be calculated for any group of patient‐age data points by dividing the number of data points with fractures by the total number of data points in the group (ie, treated group fracture probability = number of data points from treated patients with fractures divided by all data points from treated patients). For each patient‐age data point with a reported fracture, we also noted the number of fractures. Unlike fracture probability and fracture number, scoliosis probability was measured over the duration of the study. Data included information on the presence or absence of scoliosis and the degree of scoliosis when available. Information regarding age of onset of scoliosis was not available.

Logistic regression models were used to test the differences in probabilities of fractures and scoliosis between untreated and treated participants. These models used regression of fracture or scoliosis occurrence on age, LS aBMD, BPN exposure, the interaction between age and BPNs, and the interaction between LS aBMD and BPNs. The coefficient of the interaction between age and BPN exposure represented the difference in the progression of fracture or scoliosis probability with age between BPN‐exposed and BPN‐naïve groups. Similarly, the coefficient of the interaction between LS aBMD and BPN indicated whether there was a different relationship between LS aBMD and fracture or scoliosis probability based on BPN exposure. This measure was included to explore whether BPN exposure provided clinical benefits that are independent of increases in LS aBMD.

We analyzed the marginal effects of BPNs on fracture and scoliosis probabilities to determine the strength of the association between exposure and changes in clinical outcomes. Coefficients for logistic regressions are maximum likelihood estimates and can be converted to marginal effects by exponentiation. BPN‐naïve group marginal effects were derived by exponentiation of the coefficient of the continuous variable under analysis (ie, age). The BPN‐exposed group marginal effects were derived by exponentiation of the sum of the continuous variable coefficient and the coefficient of the relevant interaction term (ie, age*BPNs).

Linear regression models were used to analyze the association between BPN exposure and other clinical outcome variables: fracture number, scoliosis degree, and mobility score. These models also used a regression of outcome variable on age, LS aBMD, BPN exposure, and the two interaction terms described above.

## Results

The individuals enrolled in the LCRCs and the status of BPN exposure are listed in Table 1.

### Validating baseline assumptions

**Figure 1:**
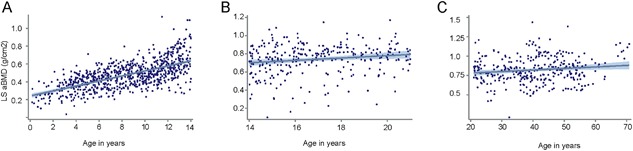
Lumbar spine areal bone mineral density (LS aBMD) versus age in osteogenesis imperfecta type I (OI‐I) in the Osteogenesis Imperfecta Foundation's linked clinical research centers . Each dot represents one single age‐LS aBMD data point. (*A*) In patients under 14 years of age, there was a strong positive relationship between LS aBMD and age (*R* ^2^ = 0.41). However, as expected, the strength of this relationship was not observed during (*B*) the later years and (*C*) adulthood.

There was a strong linear relationship between LS aBMD and age in preadolescent years (<14 years). As expected, this relationship weakened over the whole age group (Fig. 1 *A*–*C*). The age distribution of the sample was skewed toward pediatric ages and preadolescent individuals who accounted for 55% of the data points. Individuals with OI had lower LS aBMD than age‐ and gender‐specific controls (*p* < 0.01), with the mean *Z*‐score for the entire cohort being −2.04 (SD; Supplemental Fig. 1).

### Association between BPN exposure and LS aBMD in OI‐1

**Figure 2:**
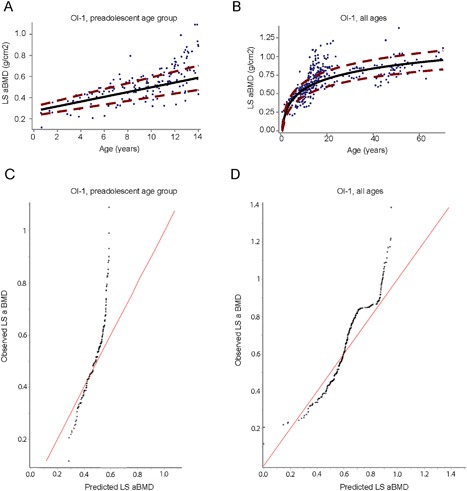
Association between bisphosphonate (BPN) use and lumbar spine areal bone mineral density (LS aBMD) in osteogenesis imperfecta type I (OI‐I). (*A*, *B*) curves represent the predicted progression of LS aBMD with age in BPN‐naïve participants. The mean expected curve (solid black line) and 95% CI (dash, red line) are depicted for the BPN‐naïve group. For participants less than 14 years of age (*A*), the predicted model is linear whereas when including all ages (*B*) the relationship is logarithmic. The blue dots represent LS aBMD values for participants who had received treatment with BPN. Dots that fall outside of the red dashed lines indicate that the treated participant LS aBMD values significantly differ from the expectations based on curves generated from individuals who were BPN naïve. (*C*, *D*) Depict quantile‐quantile plots comparing observed LS aBMD of treated participants versus predicted LS aBMD based on data from BPN‐naïve participants. (*C*) Shows that observed LS aBMD values in participants less than 14 years of age are greater than expected LS aBMD predicted from the treatment‐naïve group. (*D*) This skew is less evident in the all ages cohort.

In the preadolescent (0 to 14 years) age group in individuals with OI, BPN exposure associated with LS aBMD was 9% higher than the expected predicted value for their age and sex based on data from BPN‐naïve individuals (*p* < 0.01). However, there was no significant difference between the BPN‐exposed and BPN‐naïve groups when analysis was expanded to include all OI‐1 individuals (Fig. 2 *A*–*C*. In the preadolescent age group with OI‐1, BPN exposure was associated with higher LS aBMD relative to those who had never received BPNs (*p* < 0.01), but no such difference was observed across the entire cohort (Table 2).

**Table 2:** Association Between Type and Duration of BPN Exposure and LS aBMD in OI‐1

Ages <14 years		Any BPN	i.v. BPN	Oral BPN	>24 months of any BPN	<24 months of any BPN
Model	1	2	3	4	5	6
Age	0.031 (0.027–0.035)***	0.022 (0.016–0.028)***	0.027 (0.021–0.033)***	0.032 (0.028–0.036)***	0.024 (0.02–0.028)***	0.031 (0.027–0.035)***
						
BPN indicator		−0.071 (−0.138–−0.004)**	−0.079 (−0.146–−0.012)**	0.074 (−0.108–0.256)	−0.046 (−0.117–0.025)	−0.039 (−0.141–0.063)
						
Age*BPN [2]		0.013 (0.005–0.021)***	0.014 (0.006–0.022)***	−0.009 (−0.027–0.009)	0.012 (0.004–0.02)***	−0.003 (−0.015–0.009)
Observations	317	317	317	317	317	317
*R* ^2^	0.457	0.491	0.499	0.461	0.511	0.511
All ages						
Model	1	2	3	4	5	6
Age	0.201 (0.187–0.215)***	0.212 (0.185–0.221)***	0.202 (0.186–0.218)***	0.202 (0.188–0.216)***	0.205 (0.189–0.221)***	0.199 (0.185–0.213)***
						
BPN indicator		0.067 (−0.024–0.128)***	0.007 (−0.075–0.089)	0.003 (−0.134–0.14)	0.075 (−0.007–0.157)*	−0.047 (−0.163–0.069)
						
Age*BPN		−0.001 (−0.032–0.022)	0.014 (−0.015–0.043)	−0.004 (−0.047–0.039)	−0.011 (−0.04–0.018)	0.014 (−0.025–0.053)
Observations	670	670	670	670	670	670
*R* ^2^	0.566	0.573	0.574	0.566	0.575	0.566

The beta coefficients and the 95% CI are presented. Models 2 to 6 included a binary variable for BPN exposure. In the all ages models, log‐transformed age is used instead of chronological age. Regression model 1 uses the sample of OI‐1 patients under age 14 (top panel) or all OI‐1 patients (bottom panel) and includes only age as an explanatory variable to show the association between age and LS aBMD. Regression models 2 to 6 use the same sample, but add variables for BPN exposure and differ only in the inclusion criteria for the treated group. Regression model 2 uses participants who had received any BPN treatment. Regression model 3 uses only participants who had received i.v. BPN. Model 4 uses only participants who had received oral BPN. Model 5 uses participants treated with any BPN modality for >24 months. Model 6 uses participants treated with any BPN modality for <24 months. The Age*BPN coefficients differ significantly between models 3 and 4 (i.v. versus oral BPN; *F* = 7.58, *p* = 0.0006), as well as between models 5 and 6 (BPN >24 months versus BPN <24 months; *F* = 4.36; *p* = 0.0136). Differences between these coefficients remain significant even after Bonferroni corrections (for each model, the corrected *p* value = 0.025, *α* = 0.05).

BPN = bisphosphonate; LS = lumbar spine; aBMD = areal bone mineral density; OI‐1 = type I osteogenesis imperfecta.

*p < 0.1.

**p < 0.05.

***p < 0.01.

The difference observed between BPN‐exposed and BPN‐naïve groups was primarily driven by i.v. BPN. Exposure to oral BPN had a smaller and less significant association with LS aBMD over both the preadolescent OI‐1 age group and the whole OI‐1 cohort (Table 2; Supplemental Fig. 2). In the preadolescent ages, when compared with expected values based on participants untreated with BPN, individuals with OI‐1 who were treated with an oral BPN had a mean increase of 4% in LS aBMD (*p* < 0.05), whereas with i.v. BPN, the mean increase in LS aBMD was 10% (*p* < 0.01). BPN exposure of 24 months or greater was associated with an increase in LS aBMD above expected values when compared with exposure of less than 24 months (Supplemental Fig. 3). In the preadolescent age group, LS aBMD in individuals treated for less than 24 months did not differ significantly from the expected values, whereas in the group treated for more than 24 months’ duration, the mean density was 13% more than the expected values (*p* < 0.01). There was no significant correlation between age and duration of treatment with either oral or i.v. BPN. However, the mean duration of treatment for individuals who had received only i.v. BPN (77 months) was greater than the mean duration when treatment was with only oral BPN (30 months). Regression coefficients, representing the relationship between i.v. BPN and age, differed significantly from oral BPN versus age, as did coefficients for BPN <24 months and age versus BPN <24 months and age (Table 2). However, such differences were not observed in individuals over 14 years of age. Both males and females in the preadolescent age group who were treated with BPNs demonstrated comparable LS aBMD (9% and 10% above expectations based on individuals not treated with BPNs; *p* < 0.01; Supplemental Fig. 4).

The direction, magnitude, and statistical significance of these results were robust to all plausible alternate definitions of the “preadolescent” period, ranging from 0 to 10 years to 0 to 16 years of age. All of the above‐mentioned results were similar even when individuals who had been exposed to both oral and i.v. BPNs were excluded from the primary analysis; the only exception was that the association between oral BPNs and higher BMD lost statistical significance because of a smaller sample size.

### LS aBMD *Z*‐scores in other types of OI

**Figure 3:**
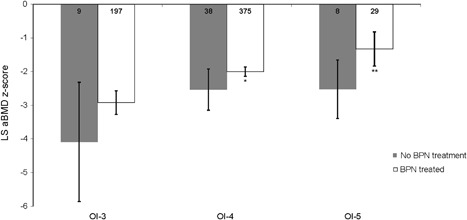
lumbar spine areal bone mineral density (LS aBMD) *Z‐*scores in osteogenesis imperfecta (OI) type III, IV, and V. The mean LS aBMD *Z‐*scores in OI‐3, OI‐4, and OI‐5 are shown. The numbers within the bars represent the sample size for each category. The error bars represent 95% CI. Asterisks next to subtype categories represent the significance of the mean difference between treated and nontreated groups for that subtype. *p < 0.1; **p < 0.05.

The mean LS aBMD *Z*‐scores in OI‐3, OI‐4, and OI‐5 with BPN exposure were −2.9, −2.0, and −1.33, respectively, as compared with −4.0, −2.5, and −2.5 in individuals who were never exposed to BPNs within the same subtypes. BPN exposure was associated with significantly higher mean LS aBMD *Z*‐scores in OI‐4 and OI‐5 as compared with individuals who had not received any treatment (Fig. 3). OI‐6 and OI‐7 could not be included in this analysis because there were no treatment‐naïve individuals (Table 1). However, even with treatment, LS aBMD *Z*‐scores in individuals with OI‐4 and OI‐5 continued to be low, with only 4% of BPN‐treated participant‐age data points having *Z*‐scores above zero.

### BPN exposure and fracture probability in OI‐1

**Figure 4:**
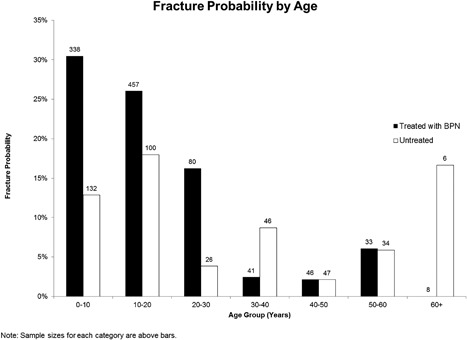
Fracture probability by age in osteogenesis imperfecta type I (OI‐I). Fracture probability categorized by age groups has been depicted. Each patient‐age data point was categorized into age groups by rounding to the nearest whole‐year age. The fracture probability decreases with age, with a significant decrease after the age of 30 years. The numbers above each bar graph depicted the sample size in each category. Note that the higher fracture probability in the treated group is likely because of ascertainment bias wherein individuals with more severe manifestations are likely to be treated with bisphosphonates.

Using the logistic regression, we demonstrate that fracture probability decreased with increases in age and LS aBMD over the entire OI‐1 cohort (Table 3, Fig. 4, Supplemental Table 2). BPN exposure had a significant association with lower fracture probability over time in the preadolescent age group. For a 1‐year increase in age in this age group, a decrease in fracture probability of 8% was observed in participants who had been treated with BPNs, whereas no such decrease was observed in participants who were treatment naïve (*p* < 0.05). Across the entire OI‐1 cohort, a 1‐year increase in age was associated with a 2% decrease in fracture probability in the untreated group (*p* < 0.05) and a 6% decrease in the treated group (6%; *p* < 0.1). In the preadolescent age group, the association of BPN exposure with lower fracture probabilities worked through mechanisms other than an increase in LS aBMD; for a 0.1 g/cm^2^ increase in LS aBMD, we found no significant change in fracture probability for untreated patients, whereas the same LS aBMD increase was associated with a 24% decrease in fracture probability in the treated group (*p* < 0.05). However, these associations (between BPNs and age as well as BPNs and LS aBMD) lost statistical significance when tested together in the same model (Supplemental Table 2). It should be noted that overall, the fracture risk is much higher in preadolescents with OI compared with adults.^21^ The association of fracture probability and BPN exposure may thus be harder to demonstrate in adults because of the decrease in fracture probability with age. Patients ever‐exposed to BPN had higher fracture probabilities up to age 30 than BPN‐naïve individuals, which likely reflects preexisting inferior bone strength in the treated group rather than any causative effect of BPN.

**Table 3:** Marginal Effects of Bisphosphonate Treatment on Other Clinical Outcome Parameters

	Ages <14 years		All ages	
Outcome variable	Untreated	Treated	Untreated	Treated
Effect of 1‐year aging (2)				
Fracture probability	8.87	−8.24**	−2.47**	−6.01*
Fracture number	0.03	−0.04**	−0.01**	−0.01*
Scoliosis probability	36.34***	0.30***	0.40	−0.80
Scoliosis degree	1.07***	0.23	−0.03	−0.21***
Mobility score	0.19***	0.43***	0.00	0.00
Effect of 0.1 g/cm^2^ BMD increase (3)				
Fracture probability	19.36	−24.26**	−10.66	−26.60
Fracture number	0.06	−0.07*	−0.03*	−0.08**
Scoliosis probability	46.76**	−10.56***	7.00	−3.99
Scoliosis degree	1.32	−1.96***	0.06	−1.36***
Mobility score	0.37**	0.75*	0.08	0.27**

(2) Values shown in the top half of the table model the changes in outcome variables for a 1‐year increase in age when other variables are constant. (3) The values in the bottom half are the changes in outcome variables for a 0.1 g/cm^2^ increase in lumbar spine areal bone mineral density. For the fracture and scoliosis probability, outcome variables are given in terms of percentage point change. For fracture number, scoliosis degree, and mobility score, the absolute numerical change is provided. The asterisks in the treated groups indicate a statistically significant difference relative to the untreated group, whereas the asterisks in the untreated groups indicate statistically significant difference from zero.

*p < 0.1.

**p < 0.05.

***p < 0.01.

### BPN exposure and fracture number in OI‐1

Using a linear regression model with the same covariates as the logistic model used to study fracture probability, we found that BPN exposure was statistically associated with fewer fractures over time in both the prepubescent and the entire cohort. However, the magnitude of these associations was too small to have any clinical implications. For example, for a 1‐year increase in age, the model predicted 0.04 fewer fractures for a participant in the BPN‐exposed preadolescent group, but no change in fracture number in the BPN‐naïve group (Table 3; *p* < 0.05). This association weakened when extended to the entire cohort (Supplemental Table 2).

### BPN use and scoliosis in OI‐1

The probability of scoliosis was highest in the age group of 10 to 20 years (Supplemental Fig. 5). Logistic regression analysis of the preadolescent age group showed that increasing age and LS aBMD values were associated with a higher scoliosis probability (Supplemental Table 2). However, participants treated with BPNs in the preadolescent age groups had a smaller increase in scoliosis probability with a 1‐year increase in age relative to participants who had not received BPNs (36% higher scoliosis probability in untreated group, 0.3% higher in treated group; Table 3; *p* < 0.01). Moreover, BPN exposure was associated with lower scoliosis probability independent of LS aBMD changes; for a 0.1 g/cm^2^ increase in LS aBMD, we found 47% higher scoliosis probability in the untreated group, but 11% lower scoliosis probability in the treated group (Table 3; *p* < 0.01). The association between BPN exposure and scoliosis probability over time in preadolescent ages remained significant when all covariates were tested together (Supplemental Table 2; *p* < 0.05). There were no significant associations between age, LS aBMD, or BPN exposure on scoliosis probability in the whole OI‐1 cohort.

### BPN use and mobility in OI‐1

Mobility was associated with age (*p* < 0.01) and higher LS aBMD values (*p* < 0.1) in the preadolescent age group (Supplemental Table 2). Treated preadolescent individuals were predicted to increase their mobility score by an average of 0.43 points with a 1‐year increase in age, relative to a 0.19‐point increase in those who were not treated (Table 3; *p* < 0.01). BPN exposure also had an impact in older individuals: For the same increase in LS aBMD (0.1 g/cm^2^), the mobility scores of treated individuals with OI‐1 increased by 0.27 on average, whereas the mobility scores of the untreated subjects over the whole cohort did not significantly change (Table 3; *p* < 0.05). This effect was also observed in the preadolescent age group, although statistical significance was weakened (Table 3; *p* < 0.1).

## Discussion

### BPN use and LS aBMD in OI‐1

Randomized placebo‐controlled trials are considered the gold standard for analyzing the effects of treatment; however, conducting such studies in rare diseases like OI is typically difficult because of the ethical and practical issues associated with randomizing individuals to a placebo arm. Moreover, BPNs, which are considered a standard treatment for OI, are medications that persist in the skeleton for years after treatment cessation. Thus, any clinical trial in OI evaluating the efficacy of a therapeutic agent has an additional challenge of accounting for previous treatment effects, even if an individual was no longer receiving BPNs at the time of enrollment.^22^ Analysis of a large data set collected from an unselected population of individuals with OI, such as the data from the OI LCRC, can be of significant value in understanding the potential benefits of BPN treatment.

Our results provide evidence that BPN exposure, past or present, is associated with higher LS aBMD values in preadolescent individuals with OI, but not in older individuals. The strongest association was observed in individuals with OI‐1; however, similar trends were also observed in individuals with OI‐4 and OI‐5. It should be noted that these results are different from many studies conducted in adults with OI that demonstrate increases in bone density with BPN treatment. Although it is well known that bone in younger subjects with OI is much more responsive to BPN treatment, many previous studies have shown that bone density increases with BPN in adults with OI.^11^, ^23^, ^24^, ^25^, ^26^ The lack of a significant association between BPN treatment and LS aBMD in older patients in our study could at least in part be based on the wide range of age distribution, type of BPN used, and duration of treatment. Treatment with oral BPNs was more common among older individuals than in the preadolescent age group, with 55% of those 14 years old and older receiving oral treatment compared with 17% of treated individuals in the 0 to 14 age group. However, a differential effect of BPN on children relative to adults is likely related to the differences in bone modeling and remodeling cycles in a growing versus mature skeleton.

### Impact of oral versus i.v. BPN treatment in OI‐1

Our analyses found that the use of i.v. BPN was more strongly associated with higher LS aBMD. Although the strongest effect of therapy is most likely because of the antiresorptive function of the drug itself, other variables could explain the difference in potency between the i.v. and oral formulations of BPN. This could be because of increased compliance with i.v. BPN. Alternatively, the small number of individuals treated with oral BPNs could influence the strength of this association. Lastly, oral treatment doses used to date may not be equivalent to the i.v. doses.

### Other potential benefits of BPN treatment in OI‐1

We demonstrate that BPN treatment was also associated with lower fracture probability, fracture number, scoliosis probability, and mobility. Although the magnitude of the associations between BPN treatment and fracture number, scoliosis degree, and mobility is small, it does provide preliminary evidence that BPNs have an impact beyond BMD. For mobility scores, even small improvements may have tangible benefits for quality of life and performing activities of daily living.

The finding that BPN treatment is associated with clinical outcomes other than BMD underscores the importance of bone quality in bone health, particularly with respect to fractures. The amount of bone mass, represented by the LS aBMD measure, is only one determinant of fracture risk. Bone quality is a crucial component in predicting fracture susceptibility; better bone architecture, bone cortex thickness and spacing, and the material properties of bone (mineralization, collagen maturity, collagen cross‐links, etc.) have an influence on the fracture risk. The association between BPN use and better clinical outcomes may be underestimated in our analyses. Because individuals with OI who are treated with BPNs may engage in more physically demanding activities, they may experience different types of fractures based on higher trauma; this is not accounted for in the current analysis.

### Limitations of the study

The analyses presented here must be interpreted within the context of the limitations of the data set. First, the dates of BPN treatment were not collected; instead, only the length of treatment was recorded. This precluded any analysis of differential BPN efficacy by age of treatment initiation. Second, the true pubertal status of patients was not collected and could be a confounding factor in the analyses. Third, BPN treatment was used as a binary variable on an individual level. The effects of BPNs are likely to be different if the treatment was recent versus being remote. This could not be included in the analyses. Fourth, fracture data were self‐reported and could not be confirmed by radiographs. Similarly, scoliosis data were obtained by various methods including radiography, medical records, and self‐reporting. Fifth, self‐reported mobility data were prone to clustering because of the scoring system of the mobility test, which showed a ceiling effect. Most mobility scores recorded were a multiple of 3 between 0 and 18, suggesting that the three mobility subscores were all rated equally for many participants (Supplemental Fig. 6). Because an individual's method of movement (walking, crutches, wheelchair, etc.) for a distance of 5 meters is inherently correlated with one's ability to move 50 meters and 500 meters, it is likely that many participants navigated each of these distances similarly. Moreover, many participant‐age data points had the maximum mobility score of 18. The clustering at 18 came from the large proportion of individuals with OI‐1 in the cohort, many of whom were scored as independently mobile at all distances. A more discriminative mobility test would be helpful in compiling more evidence for an association between BPNs and mobility improvement. Sixth, adverse events were not systematically collected; therefore, we cannot comment on the safety profile of the medications in this study. Seventh, the reading of LS aBMD was not centralized and the data were collected on different machines that were available locally at the sites; the percent changes in aBMD were thus more variable. However, individual patients with multiple data points would have had all readings done on the same DXA machine. Finally, information regarding the rationale for initiation or continuation of BPN treatment was not available. The clinical characteristics of patients before the start of BPN treatment were not available; ascertainment of such data, especially in individuals who received treatment in the years prior to enrollment in the study, was very challenging. The two groups thus could not be matched with a propensity score analysis. It is possible that the differences found between the BPN and treatment‐naïve groups may be in part because of preexisting differences between the groups themselves rather than solely based on BPN‐exposure status.

### Areas for further investigation

This study provides strong evidence that BPN treatment is associated with increased LS aBMD in preadolescent individuals with OI. This study also presents preliminary evidence that i.v. BPN treatment and longer treatment durations are associated with increases in LS aBMD and decreases in fracture incidence, scoliosis probability, and mobility. The differential effects of time of initiation of BPNs and recent versus remote treatment with BPNs on aBMD and other clinical outcomes could be assessed in future studies. Though evidence of the association between BPN treatment and increased LS aBMD is presented for other OI types, the principal analysis was limited to OI‐1. Further investigation is needed to investigate these associations for other OI types.

## Conclusion

This study provides evidence that past or present BPN exposure is associated with higher LS aBMD values in preadolescent individuals with OI. It also adds a new dimension to the OI literature by analyzing outcome variables other than LS aBMD, including fractures, scoliosis, and mobility. Our finding that BPN exposure was associated with low fracture numbers and high mobility scores when controlling for LS aBMD requires follow‐up research. Future work conducted by the NIH Rare Disease Clinical Research Network's Brittle Bone Disorders Consortium might expand on the results of this study.

## Disclosure

There are no direct conflicts of interest in the presentation of this work.

## Supporting information

Supporting Data S1.

Supporting Figures S1.

## References

[1] Forlino A , Marini JC . Osteogenesis imperfecta. Lancet (London, England). 2016;387:1657–71. 10.1016/S0140-6736(15)00728-X PMC7384887 26542481

[2] Marom R , Lee YC , Grafe I , Lee, B. Pharmacological and biological therapeutic strategies for osteogenesis imperfecta. Am J Med Genet C Semin Med Genet. 2016;172;367–83. 27813341 10.1002/ajmg.c.31532 PMC11955151

[3] Sillence DO , Senn, A , Danks DM. Genetic heterogeneity in osteogenesis imperfecta. J Med Genet. 1979;16:101–16. 458828 10.1136/jmg.16.2.101 PMC1012733

[4] Thomas IH , DiMeglio LA. Advances in the classification and treatment of osteogenesis imperfecta. Curr Osteoporos Rep. 2016;14:1–9. 26861807 10.1007/s11914-016-0299-y

[5] Patel RM , Nagamani SC , Cuthbertson D , et al. A cross‐sectional multicenter study of osteogenesis imperfecta in North America—results from the linked clinical research centers. Clin Genet. 2015;87:133–40. 24754836 10.1111/cge.12409 PMC5529599

[6] Glorieux FH , Bishop NJ , Plotkin H , Chabot N , Lanoue G , Travers R. Cyclic administration of pamidronate in children with severe osteogenesis imperfecta. N Engl J Med. 1998;339:947–52. 9753709 10.1056/NEJM199810013391402

[7] Rauch F , Glorieux FH . Osteogenesis imperfecta. Lancet. 2004;363:1377–85. 15110498 10.1016/S0140-6736(04)16051-0

[8] Ward LM , Rauch F , Whyte MP , et al. Alendronate for the treatment of pediatric osteogenesis imperfecta: a randomized placebo‐controlled study. J Clin Endocrinol Metab. 2011;96:355–64. 21106710 10.1210/jc.2010-0636

[9] Brizola E , Shapiro JR. Bisphosphonate treatment of children and adults with osteogenesis imperfecta: unanswered questions. Calcif Tissue Int. 2015;97:101–3. 26071113 10.1007/s00223-015-0021-6

[10] Chevrel G , Schott AM , Fontanges E , et al. Effects of oral alendronate on BMD in adult patients with osteogenesis imperfecta: a 3‐year randomized placebo‐controlled trial. J Bone Miner Res. 2006;21:300–6. 16418786 10.1359/JBMR.051015

[11] Falk MJ , Heeger S , Lynch KA , et al. Intravenous bisphosphonate therapy in children with osteogenesis imperfecta. Pediatrics. 2003;111:573–8. 12612238 10.1542/peds.111.3.573

[12] Biggin A , Munns CF. Osteogenesis imperfecta: diagnosis and treatment. Curr Osteopor Rep. 2014;12:279–288. 10.1007/s11914-014-0225-0 24964776

[13] Åström E , Söderhäll S. Beneficial effect of long term intravenous bisphosphonate treatment of osteogenesis imperfecta. Arch Dis Child. 2002;86:356–64. 11970931 10.1136/adc.86.5.356 PMC1751119

[14] Kusumi K , Ayoob R , Bowden SA , Ingraham S , Mahan JD. Beneficial effects of intravenous pamidronate treatment in children with osteogenesis imperfecta under 24 months of age. J Bone Miner Met. 2015;33:560–68. 10.1007/s00774-014-0618-2 25319557

[15] Rauch F , Plotkin H , Zeitlin L , Glorieux FH. Bone mass, size, and density in children and adolescents with osteogenesis imperfecta: effect of intravenous pamidronate therapy. J Bone Miner Res. 2003;18:610–4. 12674321 10.1359/jbmr.2003.18.4.610

[16] Dwan K , Phillipi CA , Steiner RD , Basel D. Bisphosphonate therapy for osteogenesis imperfecta. Cochrane Database Syst Rev. 2014;7:CD005088. 10.1002/14651858.CD005088.pub3 25054949

[17] Dwan K , Phillipi CA , Steiner RD , Basel D. Bisphosphonate therapy for osteogenesis imperfecta. Cochrane Database Syst Rev. 2016;10:CD005088. 27760454 10.1002/14651858.CD005088.pub4 PMC6611487

[18] Hald JD , Evangelou E , Langdahl BL , Ralston SH. Bisphosphonates for the prevention of fractures in osteogenesis imperfecta: meta‐analysis of placebo‐controlled trials. J Bone Miner Res. 2015;30:929–33. 25407702 10.1002/jbmr.2410

[19] Bellur, S , Jain M , Cuthbertson D , et al. Cesarean delivery is not associated with decreased at‐birth fracture rates in osteogenesis imperfecta. Genet Med. 2016;18:570–6. 26426884 10.1038/gim.2015.131 PMC4818203

[20] Graham HK , Harvey A , Rodda J , Nattrass GR , Pirpiris M. The Functional Mobility Scale (FMS). J Pediatr Orthop. 2004;24:514–20. 15308901 10.1097/00004694-200409000-00011

[21] Folkestad L , Hald JD , Ersbøll AK , et al. Fracture rates and fracture sites in patients with osteogenesis imperfecta: a nationwide register‐based cohort study. J. Bone Miner Res. 2017;32:125–34. 27448250 10.1002/jbmr.2920

[22] Orwoll ES , Shapiro J , Veith S , et al. Evaluation of teriparatide treatment in adults with osteogenesis imperfecta. J Clin Invest. 2014: 124:491–8. 24463451 10.1172/JCI71101 PMC3904621

[23] Adami S , Gatti D , Colapietro F , et al. Intravenous neridronate in adults with osteogenesis imperfecta. J Bone Miner Res. 2003;18:126–30. 12510813 10.1359/jbmr.2003.18.1.126

[24] Shapiro JR , Thompson CB , Wu Y , Nunes M , Gillen C. Bone mineral density and fracture rate in response to intravenous and oral bisphosphonates in adult osteogenesis imperfecta. Calcif Tissue Int. 2010;87:12–9. 10.1007/s00223-010-9383-y 20544187

[25] Idolazzi L , Fassio A , Viapiana O , et al. Treatment with neridronate in children and adolescents with osteogenesis imperfecta: data from open‐label, not controlled, three‐year Italian study. Bone. 2017;103:144–9. 28684193 10.1016/j.bone.2017.07.004

[26] Viapiana O , Idolazzi L , Fassio A , et al. Long‐term effects of neridronate in adults with osteogenesis imperfecta: an observational three‐year Italian study. Calcif. Tissue Int. 2017;100:341–7. 28130572 10.1007/s00223-017-0236-9

