# Supplementary material for: A Multicenter Observational Cohort Study to Evaluate the Effects of Bisphosphonate Exposure on Bone Mineral Density and Other Health Outcomes in Osteogenesis Imperfecta
Source: JBMR Plus. 2019 Jan 7;3(5):e10118. doi: 10.1002/jbm4.10118 (PMC6524673; doi:10.1002/jbm4.10118)
Supplement: Supplementary file 1 — Supporting Data S1. [file JBM4-3-na-s001.docx]

**Supplemental Data**

**Supplemental Figure Legends**

**Supplemental Figure 1: LS aBMD Z-scores in OI**

LS aBMD z-scores of participants in the OI LCRC study clearly show values below expectations relative to healthy controls matched for age and gender. Panel A depicts z-scores in OI-1 while panel B depicts z-scored from all other types of OI.

**Supplemental Figure 2 – Association of IV and oral BPN on LS aBMD in OI-1**

Each dot represents a participant-age data point from individuals who had received treatment with BPN. The mean predicted LS aBMD curves (solid blue line) and the 95% CI (dash, red lines) generated from individuals with OI-1 who had not received any BPN are depicted. Panels A and B model IV BPN treatment, while panels C and D model oral BPN treatment. Note the smaller sample sizes and wider confidence intervals in panels C and D.

**Supplemental Figure 3 – Association of duration of BPN treatment on LS aBMD in OI-1**

Each dot represents a participant-age data point from individuals who had received treatment with BPN for greater than 24 months (Panels A and B) compared to less than 24 months (Panels C and D). The mean predicted LS aBMD curves (solid blue line) and the 95% CI (dash, red lines) generated from individuals with OI-1 who had not received any BPN are depicted. The number of LS aBMD-for-age points above the predicted 95% confidence interval is clearly higher with BPN treatment duration of greater than 24 months.

**Supplemental Figure 4 – Association of BPN and LS aBMD in OI-1 categorized by gender**

Each dot represents a participant-age data point from individuals with OI-1 treated with BPN categorized by gender.

**Supplemental Figure 5 – Scoliosis probability in OI-1**

Bar graphs depicting the probability scoliosis in OI-1 across various age bins have been shown. Each patient-age data point was categorized into age groups by rounding to the nearest whole-year age. The numbers above the bars depict the data points available for each category. Only individuals in whom information of scoliosis was available were included in this analysis.

**Supplemental Figure 6 – Functional Mobility Scores**

Each dot represents the functional mobility score from a patient at a particular age. Blue dots depict scores from OI-1 whereas the red dots depict all other subtypes of OI. The functional mobility score data were highly clustered due to the nature of the scoring system. Majority of individuals with a score of 18 had OI-1 whereas majority of scores with value 18 are from OI-1 patients, whereas nearly all the scores below 9 are from individuals with other subtypes of OI.

**Supplementary Table 1: Features used for diagnosis and classification of OI in the absence of biochemical or molecular studies**

**Clinical features**

| **Type** | **Inheritance** | **Severity** | **Fractures** | **Bone Deformity** | **Stature** | **DI** | **Sclerae** | **Hearing Loss** |
| --- | --- | --- | --- | --- | --- | --- | --- | --- |
| I | AD | Mild | Few to 100 | Uncommon | Normal or slightly short for family | Rare | Blue | Present in about 50% |
| II | AD | Perinatal lethal | Multiple rib fractures, minimal calvarial mineralization, platyspondyly, marked compression of long bones | Severe | Severely short stature | + | Dark blue | — |
| III | AD; rare recessive | Severe | Thin ribs, platyspondyly, thin gracile bones with many fractures, "popcorn" epiphyses common | Moderate to severe | Very short | + | Blue | Frequent |
| IV | AD | Moderate to mild | Multiple | Mild to moderate | Variably short stature | +/- | Normal to grey | Some |
| V | AD | Moderate | Multiple with hypertrophic callus | Moderate | Variable | No | Normal | No |
| VI | Uncertain | Moderate | Multiple | Rhizomelic shortening | Mild short stature | No | Normal | No |
| VII | AR | Moderate | Multiple | Yes | Mild short stature | No | Bluish | No |

**Supplemental Table 2- Regression analysis for other clinical outcome measures**

Each vertical panel of this table uses a distinct dependent variable (shown in bold at the top-left corner of each panel). For example, the dependent variable in subheading A is fracture probability. Several regression specifications are presented for each dependent variable. Independent variables, shown in the leftmost column of the table, have corresponding coefficients and standard error (in parentheses) displayed in the table. Models (1) and (2) test the effect of BPN over time, while specifications (3) and (4) use LS aBMD as an independent variable rather than age to model the effect of BPN with changes in LS aBMD. Specification (5) considers both age and LS aBMD. The Age*BPN variable models the differential increase in the dependent variable over time for a treated patient relative to an untreated patient. Similarly, the BMD*BPN variable models the differential increase in the dependent variable for a given increase in LS aBMD for an individual treated with BPN compared to an untreated individual.
