# Supplementary material for: A Multicenter Observational Cohort Study to Evaluate the Effects of Bisphosphonate Exposure on Bone Mineral Density and Other Health Outcomes in Osteogenesis Imperfecta
Source: JBMR Plus. 2019 Jan 7;3(5):e10118. doi: 10.1002/jbm4.10118 (PMC6524673; doi:10.1002/jbm4.10118)
Supplement: Supplementary file 2 — Supporting Figures S1. [file JBM4-3-na-s002.pdf]

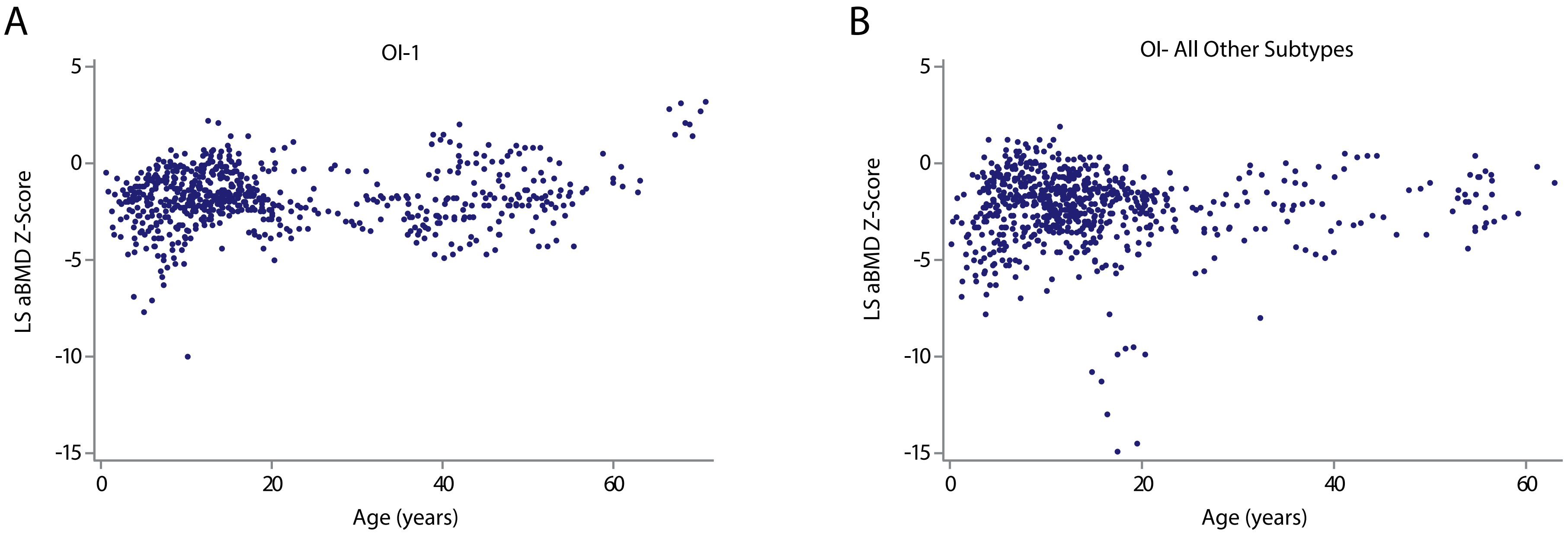


**Supplemental Figure 1**

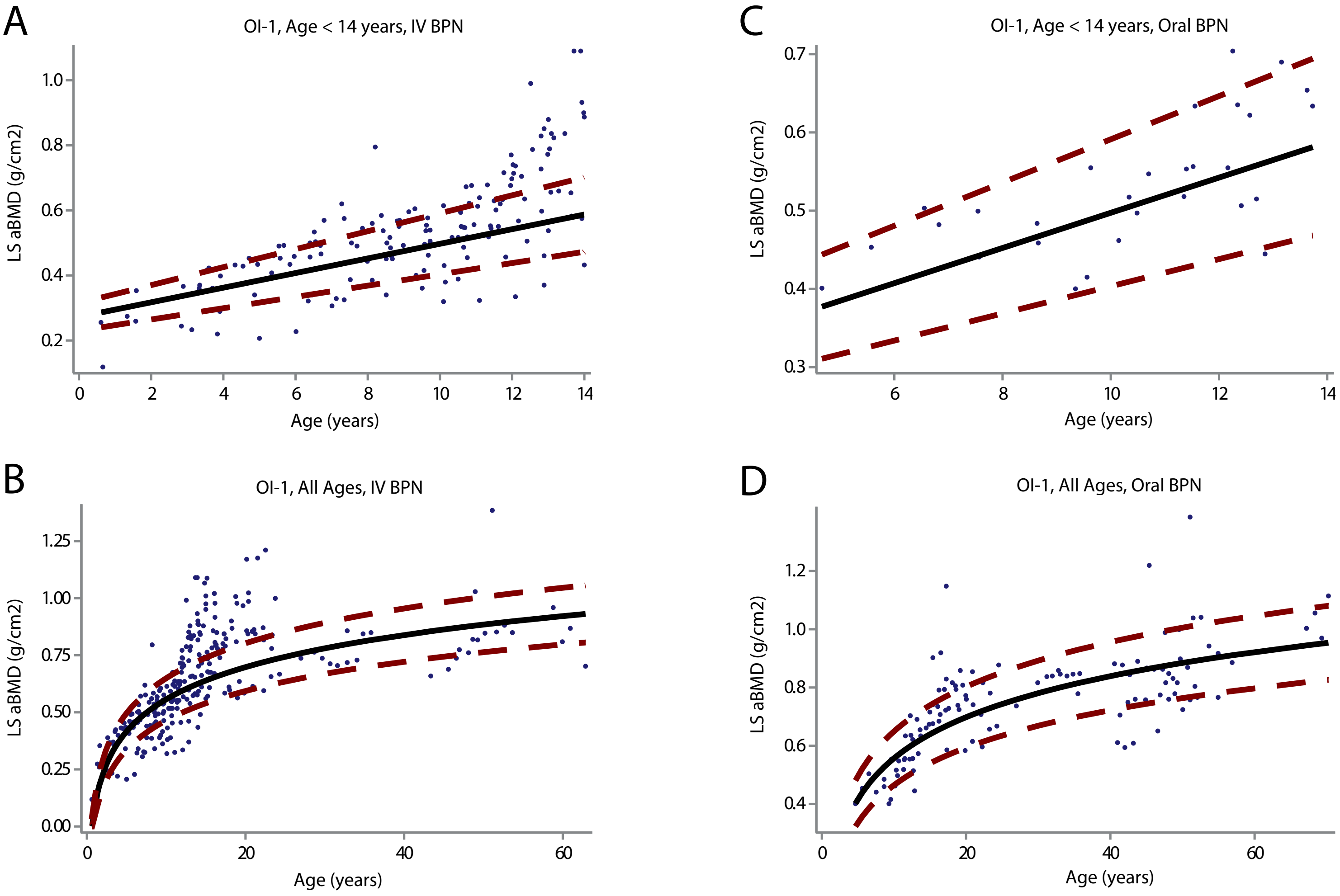


**Supplemental Figure 2**

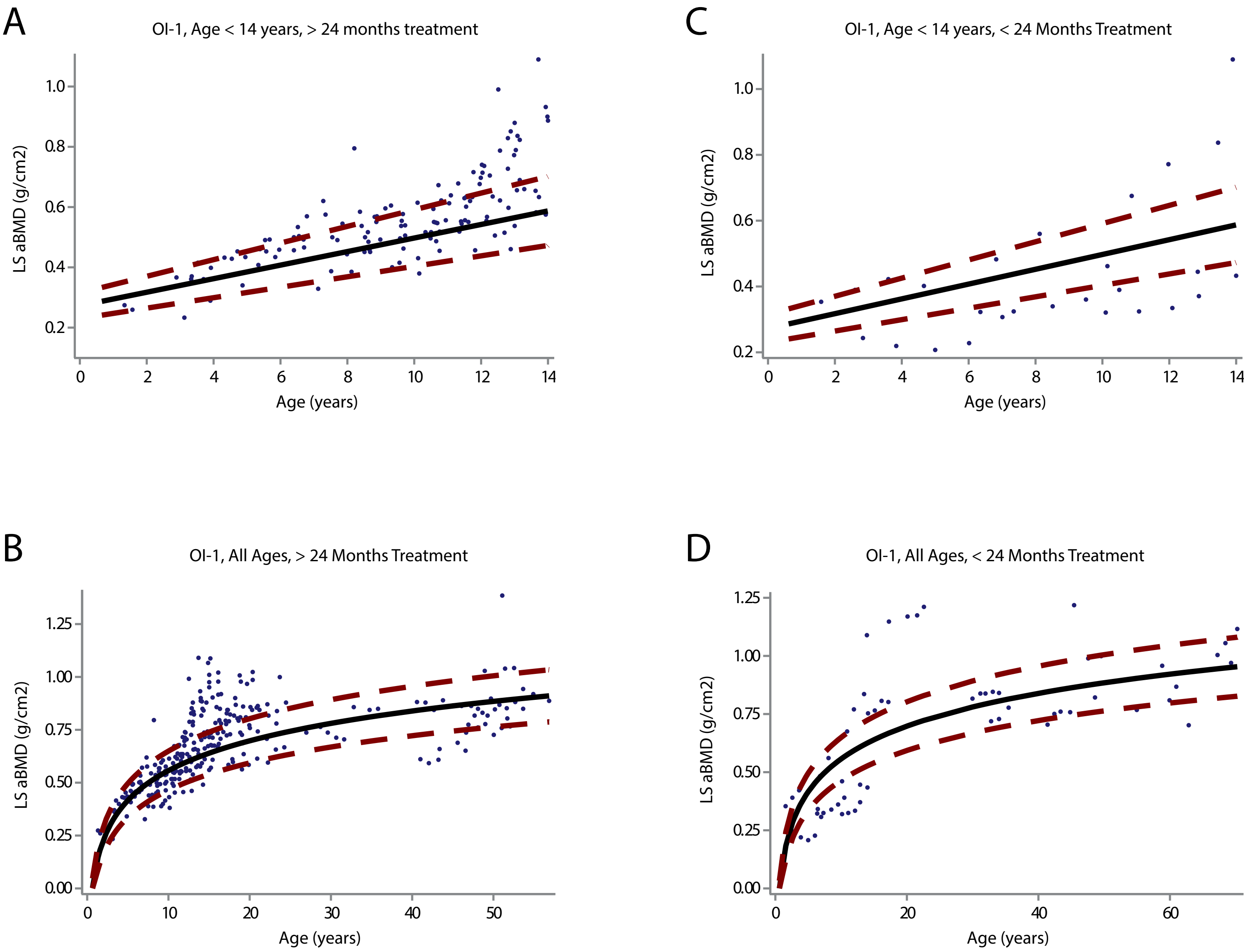


**Supplemental Figure 3**

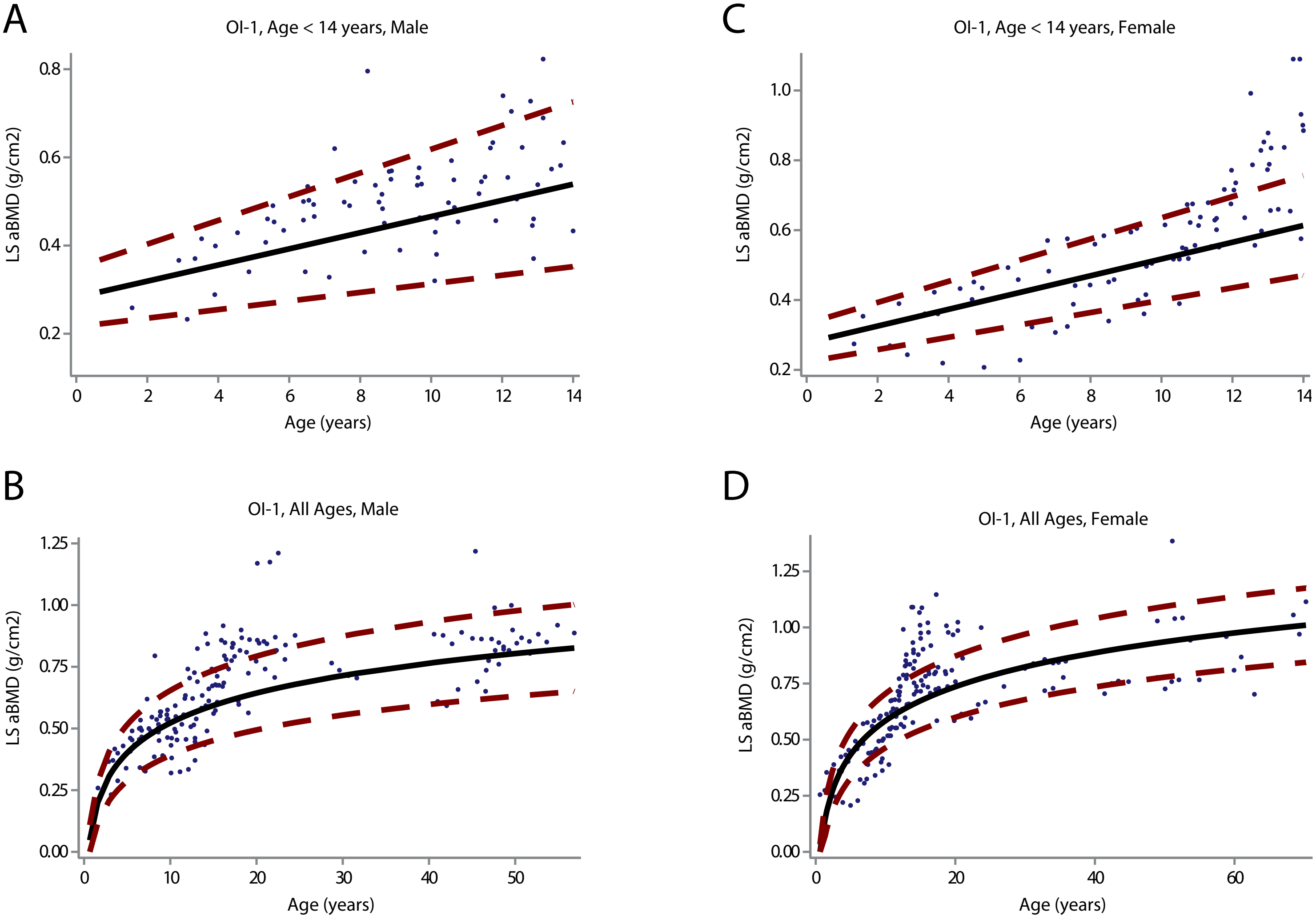


**Supplemental Figure 4**

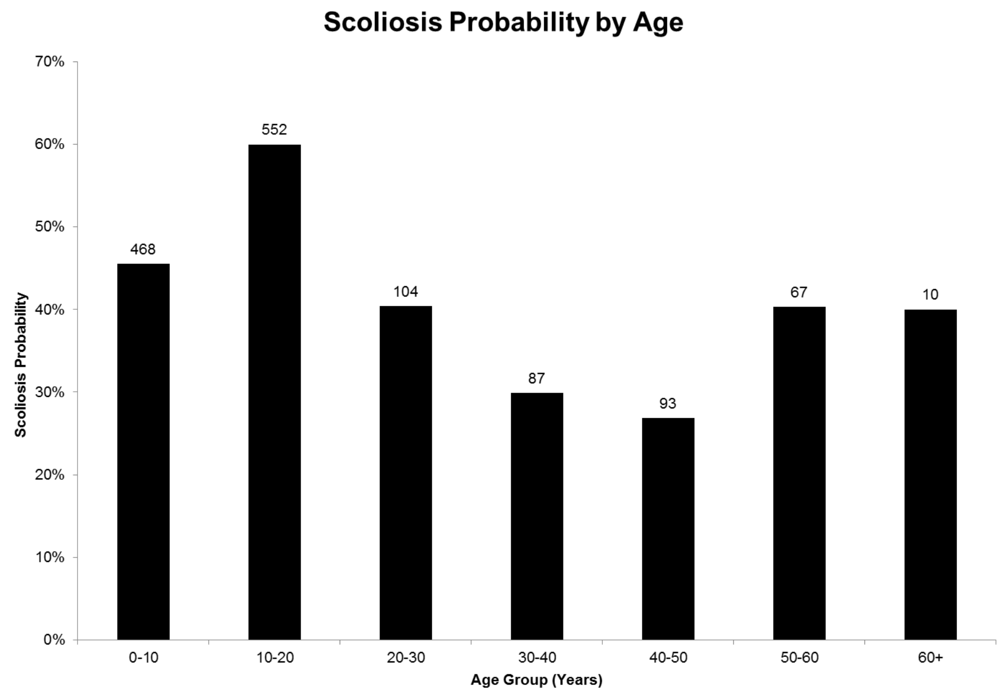


Note: Sample sizes for each category are above bars. Thirteen patient-age data points did not have information on scoliosis.

# Supplemental Figure 5

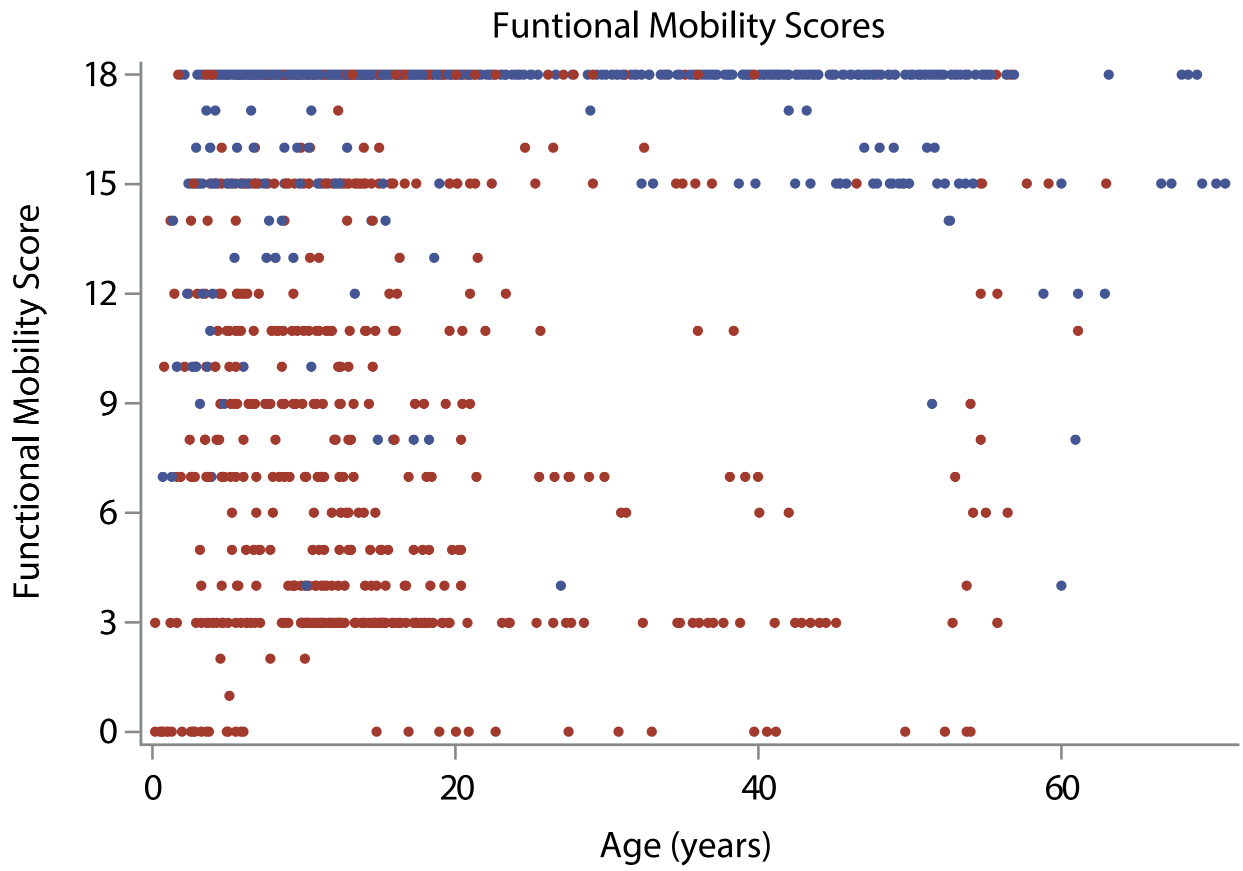


**Supplemental Figure 6**
